# THZ2 Ameliorates Mouse Colitis and Colitis-Associated Colorectal Cancer

**DOI:** 10.3390/biomedicines12030679

**Published:** 2024-03-18

**Authors:** Sheng-Te Wang, Ying-Ying Wang, Jia-Rong Huang, Yu-Bin Shu, Ke He, Zhi Shi

**Affiliations:** 1 Cancer Minimally Invasive Therapies Centre, Guangdong Second Provincial General Hospital, Jinan University, Guangzhou 510632, China; shengtewang@stu2021.jnu.edu.cn (S.-T.W.); wangyingying18@stu2021.jnu.edu.cn (Y.-Y.W.); heke8@mail3.sysu.edu.cn (K.H.); 2 Department of Cell Biology & Institute of Biomedicine, Guangdong Provincial Biotechnology & Engineering Technology Research Center, Guangdong Provincial Key Laboratory of Bioengineering Medicine, Genomic Medicine Engineering Research Center of Ministry of Education, MOE Key Laboratory of Tumor Molecular Biology, National Engineering Research Center of Genetic Medicine, State Key Laboratory of Bioactive Molecules and Druggability Assessment, College of Life Science and Technology, Jinan University, Guangzhou 510632, China; sub7@jnu.edu.cn; 3 Center for Chemical Biology and Drug Discovery, Guangzhou Institute of Biomedicine and Health, Chinese Academy of Sciences, Guangzhou 510650, China; huang_jiarong@gibh.ac.cn

**Keywords:** THZ2, CDK7, DSS, AOM, colorectal cancer

## Abstract

Colorectal cancer is a global malignancy with a high incidence and mortality rate. THZ2, a small inhibitor targeted CDK7, could inhibit multiple human tumor growths including small cell lung cancer, triple-negative breast cancer, ovarian cancer. However, the effect of THZ2 on inflammation, especially on colitis-associated colorectal cancer, is still unknown. In this study, we assessed the anti-inflammatory and anti-tumor effect of THZ2 in the mouse models of dextran sulfate sodium (DSS)-induced acute colitis and azoxymethane (AOM)/DSS-induced colitis-associated colorectal cancer. We found that THZ2 ameliorated inflammatory symptoms, including bleeding and diarrhea, in mouse models of DSS-induced acute colitis and AOM/DSS-induced colorectal cancer. The results of Western blot and immunohistochemistry showed that THZ2 rescued the up-regulated expression of COX2, IL-6, β-catenin, and snail in the mouse models. Moreover, THZ2 inhibits the development of colorectal cancer in the mouse model of AOM/DSS-induced colitis-associated colorectal cancer. Generally, THZ2 not only can inhibit DSS-induced colitis, but also can hinder AOM/DSS-induced colorectal cancer.

## 1. Introduction

Colorectal cancer is the fourth leading cause of death in cancer throughout the world, killing nearly 900,000 people each year, and can affect the quality of life through both the direct and indirect consequences of the disease [1]. Colorectal cancer accounts for about 10% of cancers diagnosed and cancer-related deaths worldwide each year. Since symptoms usually do not appear until the advanced stage, the diagnosis and treatment of colorectal cancer have become a worldwide challenge [1]. Inflammatory bowel disease (IBD), consisting primarily of Crohn’s disease and ulcerative colitis, an inflammatory disease that primarily affects the bowel, is often considered to be the prototype of inflammation-induced colorectal cancer [2]. Up to 30% of patients with ulcerative colitis develop colitis-associated colorectal cancer 35 years after the onset of the disease [3]. There was a notable rise in the incidence of colorectal cancer among patients with ulcerative colitis [4]. Effective anti-inflammatory interventions have been shown to mitigate the risk of colorectal cancer development, such as 6-mercaptopurine [5] and 5-aminosalicylic acid [6]. Anti-inflammatory therapy has demonstrated efficacy in diminishing the incidence of colorectal cancer among individuals with chronic colitis. Consequently, this treatment regimen is progressively embraced in clinical practice. Inflammation is triggered by harmful conditions such as pathogen invasion or cell damage. These events are accompanied by exposure to pathogen-associated molecular patterns or toxin-associated molecular patterns, which are perceived by pattern recognition receptors [7]. These activated receptors rapidly induce a signaling cascade that leads to the production of inflammatory mediators such as prostaglandins, and cytokines (interleukins such as IL-1, IL-6, IL-33). The mouse model of dextran sulfate sodium (DSS)-induced colitis, commonly utilized for studying colitis, is favored due to its pathogenic similarity to human colitis [8]. DSS colitis models have been widely used in IBD research because these models are indispensable tools for deciphering the pathogenesis of IBD and evaluating many potential therapies [9]. DSS is a chemical colonogen with anticoagulant properties to induce disease. DSS is a water-soluble, negatively charged sulfate polysaccharide with a molecular weight that varies greatly between 5~1400 kDa. The high negative charge generated by the sulfate group is toxic to epithelial cells of the colon and induces erosion, compromising the integrity of the barrier. DSS forms nanoscale vesicles with medium-chain length fatty acids in the colonic lumen to activate intestinal inflammatory signaling pathways [10]. DSS is administered for 3–7 days, followed by regular water administration for 1–2 weeks to allow the colonic mucosa to heal to induce colonic inflammation in mice, as observed in patients with chronic ulcerative colitis. Long-term use of DSS is employed to induce colorectal cancer [11]. Squamous metaplasia of rectal mucosa, squamous papilloma, adenoma, and adenocarcinoma are observed in DSS-treated mice [12]. The common animal model of colorectal cancer is the mouse model of azoxymethane (AOM)/DSS-induced colorectal cancer. 1-week administration of 2% DSS after initiation with a low dose of AOM exerts a powerful tumor-promoting activity in colon carcinogenesis in mice [13]. The AOM/DSS model is a powerful, reproducible, and relatively inexpensive model that induces DNA damage followed by a repeat cycle of colitis [13]. AOM, an oxide of azomethane, is a carcinogen that can be converted to methyl azoethanol, leading to a G→A transition [14]. AOM is the most widely used carcinogen in the colon to promote the formation of colorectal tumors in rodents. The AOM/DSS model is characterized by its relatively short timeline and precise replication of colorectal cancer characteristics. Tumor development can manifest within a notably brief period, as short as 10 weeks [13,15]. The histopathology of AOM/DSS-induced tumors recapitulates the abnormal crypt foci-adenoma-carcinoma sequences occurring in human colorectal cancer [16].

Cyclin-dependent kinases (CDKs) are key enzymes that regulate all cell cycle transitions and are considered as effective therapeutic targets for cancer [17]. CDK7 is a subunit of the multi-protein transcription factor TFIIH, which can participate in the transcriptional regulation of many oncogenes by phosphorylating the C-terminal domain of RNAPII [18]. It has been reported that THZ1, a specific inhibitor of CDK7 to block the phosphorylation of the CDK7 substrate RNAPII CTD by irreversible covalent binding to CDK7, can negatively affect gene expression, inhibiting the proliferation of cancer cells and the progression of animal xenograft models [19]. The short half-life time of THZ1, only 45 min, limits its application. THZ2 is an analogue of THZ1 with a five-fold increased half-life, which effectively inhibits the growth of various cancer cells by inhibiting CDK7 as potent as THZ1, including breast cancer [20] and osteosarcoma [21]. However, the effect of THZ2 on inflammation, especially on colitis-associated colorectal cancer, is still unknown. In this study, we assessed the anti-inflammatory and anti-tumor effect of THZ2 in the mouse models of DSS-induced acute colitis and AOM/DSS-induced colitis-associated colorectal cancer respectively.

## 2. Materials and Methods

### 2.1. Reagents 

Azoxymethane (AOM) (CAS No: 25843-45-2) was obtained from Sigma-Aldrich (Shanghai, China). Dextran sodium sulfate (DSS) (CAS No: 9011-18-1) was purchased from Yeasen Biotechnology Co., Ltd. (Shanghai, China). THZ2 (CAS No: 1604810-84-5) was purchased from ApexBio Technology (Houston, TX, USA). Anti-IL-6 antibody (#4ab080344) was purchased from 4A Biotech Co., Ltd. (Beijing, China). Anti-COX-2 antibody (#BA0738) was purchased from BOSTER Biological Technology Co., Ltd. (Pleasanton, CA, USA). Anti-β-tubulin antibody (#KM9003T) was sourced from Tianjin SUNGENE BIOTECH Ltd. (Tianjin, China). Anti-β-Catenin antibody (#610154) was purchased from BD Biosciences (Franklin Lakes, NJ, USA). Anti-snail antibody (#RLT4351) was purchased from Shandong Ruiying Pharmaceutical Group Co., Ltd. (Shandong, China).

### 2.2. Mice

8-week-old BALB/c male mice were purchased from the Guangdong provincial medical laboratory animal center. Mice are fed with sterilized food and water in a pathogen-free facility. The control group comprises three mice, while the model group consists of five mice, and the THZ2 group includes five mice. All experimental procedures were approved by the Institutional Animal Care and Use Committee of Jinan University.

### 2.3. Protocol of Animal Models of DSS-Induced Acute Colitis

The experimental protocol was executed in accordance with the described procedure, with minor modifications [9]. Mice were administered drinking water containing 2.5% DSS for a period of one week to induce acute colitis, followed by switching to regular drinking water for another one week. Mice were injected with THZ2 (10 mg/kg) or solution control (0.5% methylcellulose) intraperitoneally twice every day for two weeks.

### 2.4. Protocol of Animal Model of AOM/DSS–Induced Colorectal Cancer

The establishment of a mouse model for colitis-associated colorectal cancer has been previously documented [22,23]. On the initial day, mice received intraperitoneal injections of AOM (10 mg/kg). From the second week onward, the regular drinking water provided to the mice was replaced with water containing 2.5% DSS, followed by a subsequent switch back to standard drinking water for two weeks. Mice were administered with three cycles of DSS treatment consisting of one week of 2.5% DSS treatment and two weeks of regular water. Mice were injected with THZ2 (10 mg/kg) or solution control (0.5% methylcellulose) intraperitoneally every other day for nine weeks.

### 2.5. Clinical Assessment of DSS-Induced Acute Colitis and AOM/DSS–Induced Colorectal Cancer Mouse Models

Body weight, diarrhea degree, and the bleeding degree of rectum were measured in the mice every two days. Bleeding degree was scored as 0 for no blood, 2 for slight bleeding, and 4 for gross bleeding. Diarrhea degree was scored as 0 for well-formed pellets, 0.3 for soft pellets, 0.6 for pasty stools, and 0.9 for liquid stools.

### 2.6. Treatments and Sample Collection

Throughout the study, measurements of body weight, diarrhea, and fecal bleeding in mice were conducted every two days. Colon length is measured after the mice are sacrificed and then fixed in 10% formalin for future analysis.

### 2.7. Western Blot 

Colon tissue samples were rinsed twice with cold PBS and subjected to lysis in RIPA buffer (1% NP-40, 0.5% sodium deoxycholate, 0.1% SDS, 10 ng/mL PMSF, 0.03% aprotinin, 1 μM sodium orthovanadate) at 4 °C for 30 min. After centrifuging for 10 min at 14,000× *g*, supernatants were collected and stored at −80 °C. The protein concentration was detected with the Bradford assay. Proteins were separated on 12% SDS-PAGE gels and transferred to polyvinylidene difluoride membranes. After blocked with 5% BSA, the membranes were incubated with the specified primary antibodies at 4 °C overnight and the corresponding horseradish peroxidase-conjugated secondary antibodies for 1 h. Proteins were examined with the chemiluminescent detection reagents and Bio-Rad image system. The semi-quantitative analysis of protein bands was performed with the software Image J (https://imagej.net/ij/).

### 2.8. Histopathological Examination 

For histopathology analysis, formalin-fixed, paraffin-embedded tumor tissue slides were deparaffinized using xylene and graded ethyl alcohol. Histological staining was performed on the specimens using hematoxylin and eosin (H&E).

### 2.9. Immunohistochemistry Assay 

Immunohistochemistry assay was performed with a microwave-enhanced avidin-biotin staining method. Formalin-fixed tumor tissue slides embedded in paraffin were deparaffinized using xylene and graded ethyl alcohol, followed by water rinsing. Antigen retrieval involved boiling the slides in 0.01M citrate buffer (pH = 6) in a microwave oven for 10 min, followed by cooling to room temperature. The slides were incubated with 0.05% Triton X-100 in PBS for 5 min, followed by sequential treatments in a humidified chamber: quenching endogenous peroxides with 3% H_2_O_2_ in MeOH, blocking serum with avidin for 20 min, incubating with the primary antibody overnight at 4 °C, applying the secondary antibody for 20 min, treating with hydrogen peroxidase for 15 min, and finally, exposing to peroxidase substrate solution for 20 min at room temperature. After staining, the slides underwent counterstaining with hematoxylin and were then cover-slipped. The quantification of protein expression utilized the subsequent formula: immunohistochemical score = percentage of positive cells × intensity score. The intensity was assessed using the following scoring system: 0 for negative, 1 for weak, 2 for moderate, and 3 for intense. A cell with immunohistochemical score of ≥50 was deemed positive.

### 2.10. Statistical Analysis 

The differences were determined using the Student’s *t*-test. Data were shown as mean ± SD. The statistical significance was denoted by ‘*’ for *p* < 0.05, and ‘**’ for *p* < 0.01.

## 3. Results 

### 3.1. THZ2 Inhibits the Inflammation in DSS-Induced Acute Colitis Mouse Models

**Figure 1:**
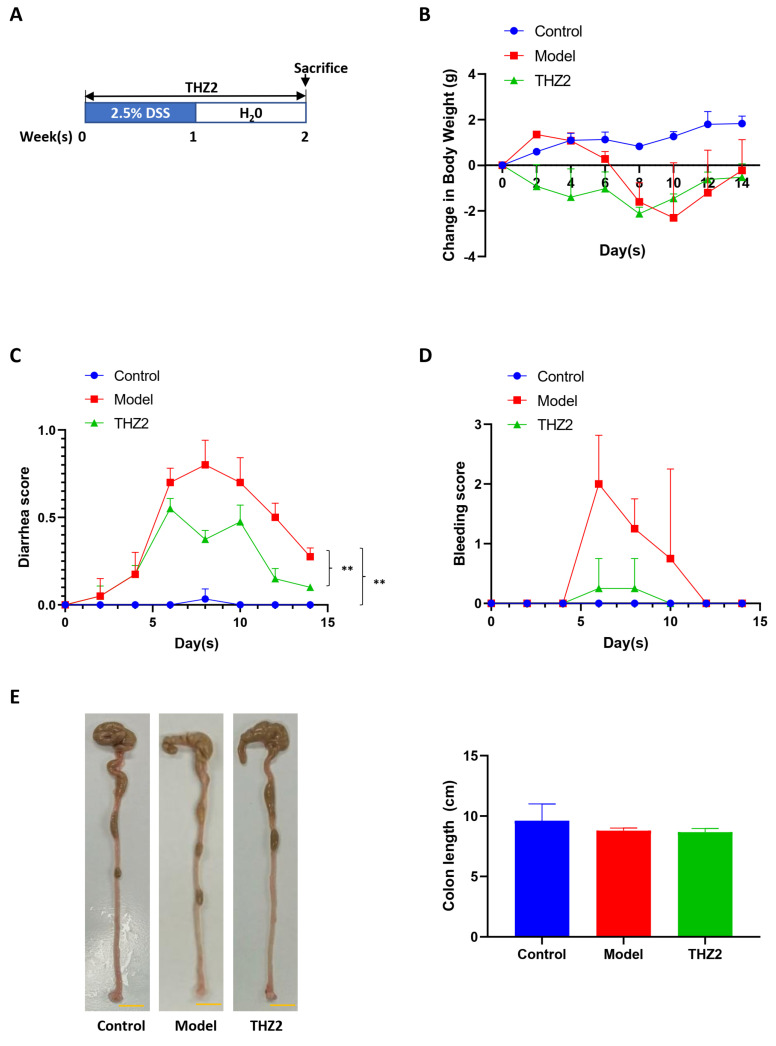
THZ2 ameliorates the inflammatory symptoms in DSS-induced acute colitis mouse models. (**A**) The Schematic diagram of DSS-induced colitis. (**B**) The change in body weight of mice. (**C**) Diarrhea score. (**D**) Bleeding score. (**E**) Representative of colon and colon length. Note: Control, no treated group; Model, DSS treated group; THZ2, THZ2 in combination with DSS-treated group. ** *p* < 0.01 vs. corresponding group.

Inhibiting inflammation of the colon and rectum is an important strategy to prevent colorectal cancer. In order to explore the effect of THZ2, a small molecule inhibitor of CDK7 on colitis, we generated acute colitis with DSS in BALB/c mice. The schematic diagram of the modeling of acute colitis in mice is shown in Figure 1A. Mice were administered drinking water containing 2.5% DSS for a period of one week to induce acute colitis, followed by switching to regular drinking water for another one week. As shown in Figure 1B–D, the mice treated with DSS showed more severe symptoms of diarrhea and bleeding compared with the control group, indicating that DSS-induced acute colitis mouse models had been successfully established. Mice were injected with THZ2 (10 mg/kg) or solution control (0.5% methylcellulose) intraperitoneally twice every day for two weeks. As shown in Figure 1B–D, there was no substantial distinction in the body weight between the THZ2 group and the model group, while THZ2 significantly alleviated the inflammatory symptoms (diarrhea and bleeding) in DSS-induced acute colitis mouse models. On day 15, the mice were euthanized and the colon was harvested to evaluate the effect of THZ2 on the colon in the DSS model. As depicted in Figure 1E, the length of the colon did not exhibit any noteworthy alterations. These data suggest that THZ2 inhibits inflammation in mouse models of acute colitis induced by DSS.

### 3.2. THZ2 Suppresses the Expression of COX-2, IL-6, β-Catenin and Snail in DSS-Induced Acute Colitis Mouse Models

**Figure 2:**
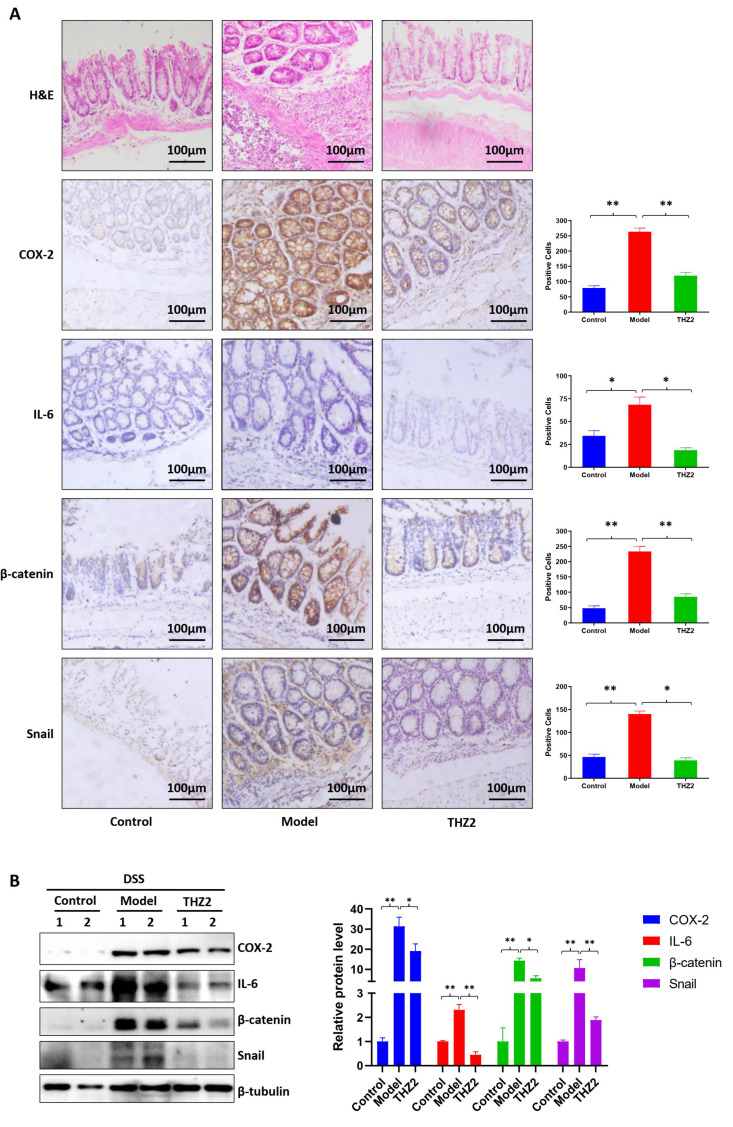
THZ2 inhibits the expression of COX-2, IL-6, β-catenin, and snail in DSS-induced acute colitis mouse models. (**A**) Representative colonic sections were immunohistochemically stained with H&E and the indicated antibodies. (**B**) The protein in colorectal tissues from two mice in each group were examined by Western blot analysis. The representative results and quantified data are shown. Note: Control, no treated group; Model, DSS treated group; THZ2, THZ2 in combination with DSS-treated group. * *p* < 0.05, and ** *p* < 0.01 vs. corresponding group.

In order to investigate whether the mechanism of THZ2 in the DSS-induced colitis model is related to the regulation of inflammatory protein, we performed the histopathological examination, immunostaining, and Western blot on mouse colon tissues to detect the expression of COX-2, IL-6, β-catenin, and snail. As shown in Figure 2A, the results of histopathological examination revealed that DSS treatment generated epithelial architecture destruction and inflammatory cell infiltration while THZ2 can prevent this effect. As shown by immunohistochemistry staining and Western blot, THZ2 also reversed the promotion of COX-2, IL-6, β-catenin, and snail by DSS in the acute colitis mouse models (Figure 2A,B). These data suggest that THZ2 inhibits the expression of COX-2, IL-6, β-catenin, and snail in mouse models of acute colitis induced by DSS.

### 3.3. THZ2 Ameliorates the Inflammatory Symptoms and Inhibits Tumor Growth of AOM/DSS-Induced Colorectal Cancer Mouse Models

**Figure 3:**
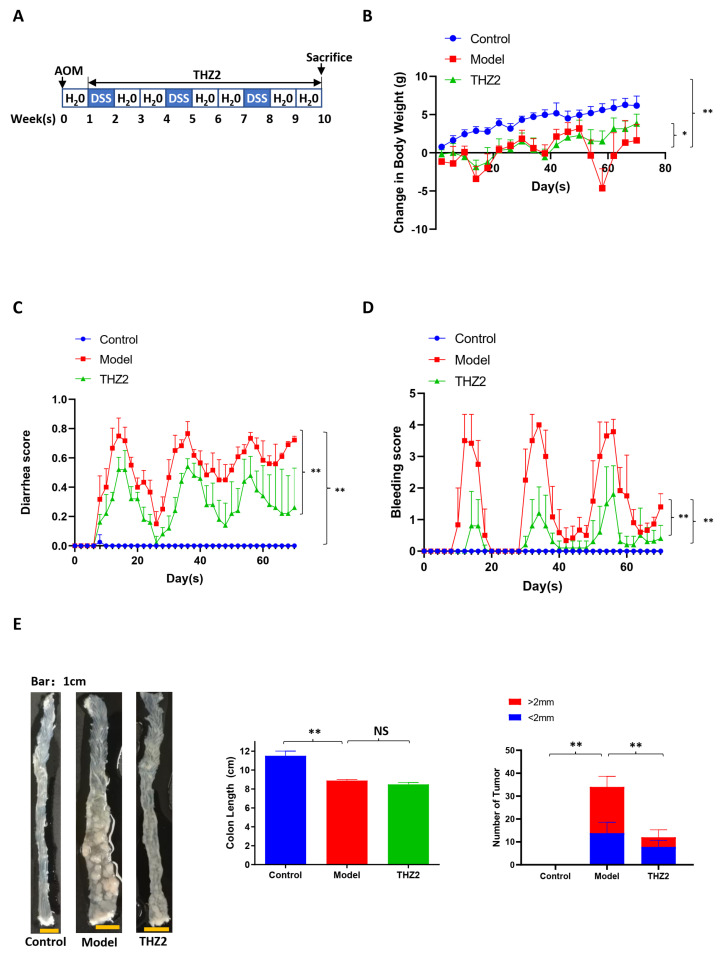
THZ2 ameliorates the inflammatory symptoms and inhibits tumor growth of AOM/DSS-induced colorectal cancer mouse models. (**A**) The schematic diagram of AOM/DSS-induced colorectal cancer. (**B**) The change in body weight of mice. (**C**) Diarrhea score. (**D**) Bleeding score. (**E**) Representative of colon and number of tumors. Note: Control, no treated group; Model, AOM/DSS treated group; THZ2, THZ2 in combination with AOM/DSS-treated group. NS, not significant. * *p* < 0.05, and ** *p* < 0.01 vs. corresponding group.

To access the anti-cancer function of THZ2 in colorectal cancer, we built the colorectal cancer mouse models induced by AOM in combination with DSS. The schematic diagram of the modeling AOM/DSS-induced colorectal cancer is shown in Figure 3A. On the initial day, mice received intraperitoneal injections of AOM (10 mg/kg). From the second week onward, the regular drinking water provided to the mice was replaced with water containing 2.5% DSS, followed by a subsequent switch back to standard drinking water for two weeks. Mice were administered with three cycles of DSS treatment consisting of one week of 2.5% DSS treatment and two weeks of regular water. Mice were injected with THZ2 (10 mg/kg) or solution control (0.5% methylcellulose) intraperitoneally twice every day for nine weeks. As shown in Figure 3B–E, compared with the control group, AOM/DSS-treated mice showed more severe diarrhea and bleeding symptoms, as well as more intestinal tumors, indicating that AOM/DSS-induced colorectal cancer mouse model has been successfully established. THZ2 can alleviate colorectal cancer symptoms induced by AOM/DSS in mice, inclusive of symptoms such as diarrhea, bleeding, and body weight loss (Figure 3B–D). No notable disparity in colon length was observed between the THZ2 group and the Model group, while THZ2 could inhibit tumor growth in the AOM/DSS-induced colorectal cancer, as is shown in Figure 3E. These data suggest that THZ2 alleviates the inflammatory symptoms and inhibits tumor growth of AOM/DSS-induced colorectal cancer mouse models.

### 3.4. THZ2 Suppresses the Expression of COX-2, IL-6, β-Catenin and Snail in AOM/DSS-Induced Colorectal Cancer Mouse Models

**Figure 4:**
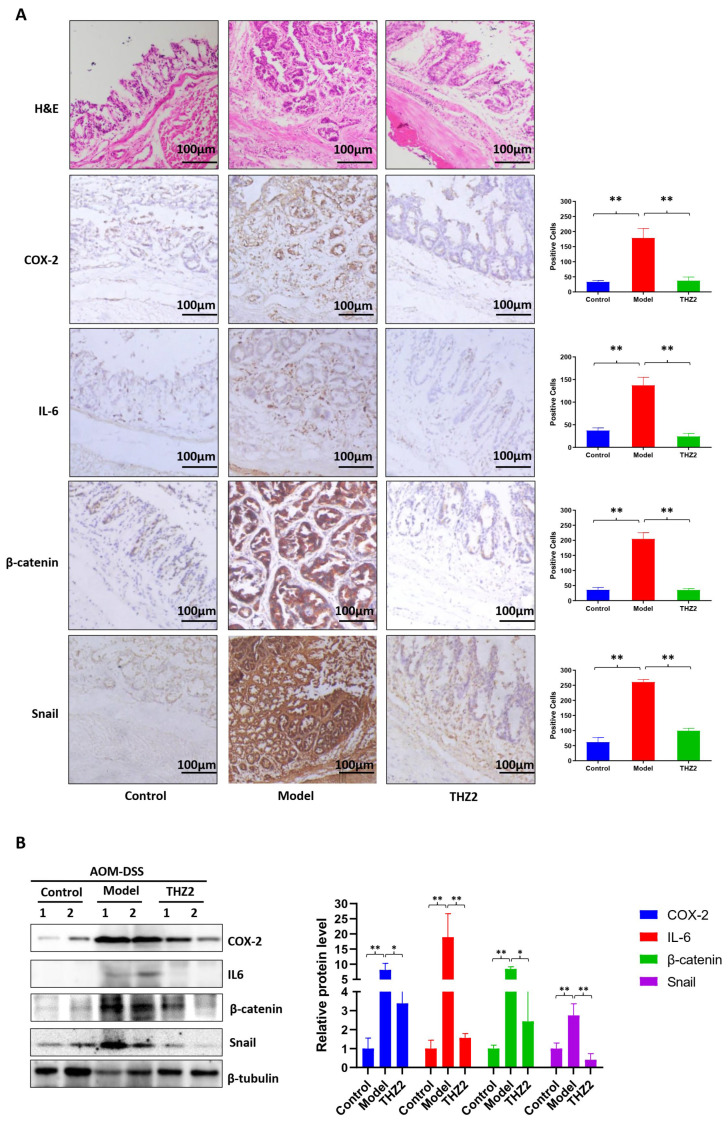
THZ2 inhibits the expression of COX-2, IL-6, β-catenin, and snail in AOM/DSS-induced colorectal cancer mouse models. (**A**) Representative colonic sections were immunohistochemically stained with H&E and the indicated antibodies. (**B**) The protein in colorectal tissues from two mice in each group were examined by Western blot analysis. The representative results and quantified data are shown. Note: Control, no treated group; Model, AOM/DSS treated group; THZ2, THZ2 in combination with AOM/DSS-treated group. * *p* < 0.05, and ** *p* < 0.01 vs. corresponding group.

In order to investigate the mechanism of THZ2 in the DSS-induced colitis model, mouse colon tissues from AOM/DSS-induced colorectal cancer mouse models were examined through histopathological examination, immunostaining, and Western blot. As is shown in Figure 4A, the results of the histopathological examination revealed that THZ2 treatment could reduce the inflammatory cell infiltration induced by AOM/DSS-induced colorectal cancer. Immunohistochemistry results indicated that THZ2 could prevent the up-regulation of the expression of COX-2, IL-6, β-catenin, and snail in AOM/DSS-induced colorectal cancer tissues. Consistent outcomes were achieved, as depicted in Figure 4B, through Western blot analysis. These data suggest that THZ2 inhibits the expression of COX-2, IL-6, β-catenin, and snail in mouse models of colorectal cancer induced by AOM/DSS.

## 4. Discussion

Consistent usage of aspirin and nonsteroidal anti-inflammatory drugs (NSAIDs) has been demonstrated to prevent colorectal polyps and is correlated with a decreased risk of colorectal cancer [24,25]. The likelihood of cancer metastasis is reduced in individuals who use aspirin [26]. The best-known target of NSAIDs is cyclooxygenase, which converts arachidonic acid into prostaglandins (PG) and thromboxanes [27,28]. Numerous data from animal models and human studies suggest that the upregulation of COX-2 in cancer cells enhances tumor progression. Conversely, NSAIDs inhibit COX-2, thereby exhibiting anti-tumor properties [29]. COX-2 exhibits a notable increase in expression within AOM-induced colon tumors in rodent models [30]. In the early stages of colorectal cancer, COX-2 undergoes upregulation, leading to the production of prostaglandins, which promote cancer cell proliferation, hinder apoptosis, and enhance angiogenesis, thereby facilitating tumor growth and metastasis [31]. IL-6 is produced primarily by macrophages exposed to specific microorganisms. IL-6 has environmentally-dependent pro-inflammatory and anti-inflammatory properties and is now considered an important target for clinical interventions [32]. As a multifunctional cytokine regulated by NF-κB, IL-6 is a key tumor promoter during early colorectal cancer development [33]. IL-6 is highly upregulated in animal models of colorectal cancer [34,35]. Similarly, elevated serum levels of IL-6 have been found in colorectal cancer patients [36]. In this study, we showed that THZ2 ameliorated the inflammatory symptoms of acute colitis and colorectal cancer, including bleeding and diarrhea. THZ2 rescued the up-expression of COX2 and IL-6 in DSS-induced colitis and AOM/DSS-induced colitis-associated colorectal cancer. Similar to our data, another CDK7 inhibitor BS-181 also inhibits the expression of IL-6 and the inflammation of rheumatoid arthritis [37].

Classical Wnt signaling plays a crucial role in regulating normal intestinal maturation and colorectal tumorigenesis [38]. β-catenin is a major player in controlling adult epithelial homeostasis in the Wnt-signaling pathway, and mutations are often present in hereditary cancers to dissolve this signaling cascade [39]. In cancer progression, cancer cells are thought to have acquired a mesenchymal phenotype, which allows them to leave the primary site, and invade surrounding tissues to form metastases [40]. The process by which cells transition from an epithelial to a mesenchymal phenotype is called epithelial-mesenchymal transition (EMT) [41]. The inflammatory microenvironment of cancer cells is also a decisive factor in the induction of pathological EMT, which can upregulate snail [42]. CDK7 has been found to activate the β-catenin pathway in hepatocellular carcinoma [43]. Our study found that THZ2 may exert its inhibitory effect on colorectal cancer development by reducing the expression of β-catenin and snail in AOM/DSS mouse models, suggesting that CDK7 could potentially serve as a therapeutic target in cancer treatment by affecting β-catenin.

CDKs are well-defined targets for inhibitors used for the treatment of cancer [44,45]. CDKs accelerate inflammation by triggering the function of pro-inflammatory transcription factors such as NF-κB, STAT3, and AP-1. CDKs can regulate gene expression by phosphorylating RNA Pol II CTD, targeting global transcription, or they can affect gene expression in a highly specific manner by directly binding to pro-inflammatory transcription factors [46]. Dysregulation of the cell cycle is a well-established characteristic in cancer, and aberrant control of transcriptional processes through multiple mechanisms is also common in many cancers. As a member of the CDKs, CDK7 is a major regulator of cell cycle progression [47]. CDK7 controls the cell cycle by phosphorylating the CDK1, 2, 4, and 6 [48]. CDK7 regulates gene expression and is involved in regulating the initiation and extension of RNA polymerase II as a component of the general transcription factor complex TFIIH [49]. CDK7 levels are elevated in many cancer types, suggesting that cancer tissues are more dependent on CDK7 activity than normal tissues [50]. CDK7 has also become a target for small-molecule inhibitors, showing promise in the treatment of cancer and inflammation [51]. CDK7i can cause cell cycle arrest, apoptosis, and transcriptional repression, especially in super-enhancer-related genes in cancer, and has demonstrated its potential to overcome cancer treatment resistance. Four CDK7i, ICEC0942 (CT7001), SY-1365, SY-5609, and LY3405105 are now in Phase I/II clinical trials [52]. Inhibition of CDK7 by BS-181 significantly hinders the development of collagen-induced arthritis in mice [37]. Inhibition of CDK7 was demonstrated using the selective CDK7 inhibitor YKL-5-124 to predominantly disrupt cell cycle progression and induce DNA replication stress and genomic instability in small cell lung cancer, while triggering immune response signaling. This effect can be significantly enhanced by combining YKL-5-124 with anti-PD-1 [53]. THZ2 can completely inhibit the phosphorylation of the intracellular CDK7 substrate RNAPII CTD at Ser-2, -5, and -7 by irreversibly covalently binding to CDK7 [54]. THZ2 is an analogue of THZ1 which is a potent and selective irreversible CDK7 inhibitor with promising anticancer activity in various cancers [55,56]. THZ2 presented outstanding anticancer effects in vitro and in vivo, with a 5-fold increased half-life over THZ1, and can inhibit triple-negative breast cancer [20]. In our previous reports, we found that THZ2 was able to suppress cell growth, arrest cell cycle at the G2/M phase, and induce cell apoptosis with enhanced intracellular levels of reactive oxidative species in gastric cancer cells. Pretreatment with ROS inhibitor N-acety-L-cysteine partially rescued THZ2-induced cell apoptosis. [57]. Moreover, the THZ2-targeted super enhancer exhibits strong anti-osteosarcoma effects both in vitro and in vivo [21]. In the present study, our results show that THZ2 inhibits the development of colorectal cancer in the mouse model of AOM/DSS-induced colitis-associated colorectal cancer. However, the use of THZ2 for human colitis and colorectal cancer treatment needs to be further investigated in the future.

## 5. Conclusions

In summary, as a prospective inhibitor of CDK7, THZ2 can alleviate the inflammatory symptoms, both in DSS-induced acute colitis and AOM/DSS-induced colorectal cancer mouse models, inhibiting colitis and tumor cell proliferation. The mechanism may be associated with the down-regulation of COX2, IL-6, β-catenin, and snail. Our data shows that THZ2 may offer a potential therapy strategy for colitis and colitis-associated colorectal cancer.

## Data Availability

Data is available upon reasonable request to corresponding author.
